# Editorial: Reviews in breast cancer: 2023

**DOI:** 10.3389/fonc.2024.1488263

**Published:** 2024-10-16

**Authors:** Daniel P. Bezerra, Jie Ni, Sulma I. Mohammed, Maria Rosaria De Miglio

**Affiliations:** ^1^ Gonçalo Moniz Institute, Oswaldo Cruz Foundation (IGM-FIOCRUZ/BA), Salvador, BA, Brazil; ^2^ School of Clinical Medicine Faculty of Medicine and Health, University of New South Wales (UNSW), Sydney, NSW, Australia; ^3^ Cancer Care Centre, St. George Hospital, Kogarah, NSW, Australia; ^4^ Purdue University Institute for Cancer Research, Department of Comparative Pathobiology, Purdue University, West Lafayette, IN, United States; ^5^ Department of Medicine, Surgery and Pharmacy, University of Sassari, Sassari, Italy

**Keywords:** breast cancer, epidemiology, review, tumor microenvironment, biomarkers

Breast cancer is a heterogeneous disease characterized by several subgroups that can be identified through molecular biomarkers, which may serve as predictive indicators (1). Globally, female breast cancer ranks as the second most common cancer, with 2,308,897 new cases diagnosed, and the fourth leading cause of cancer-related deaths, with 665,684 fatalities in 2022 (2). Furthermore, the GLOBOCAN Cancer Tomorrow prediction tool estimates that the incidence of breast cancer will increase by more than 46% by 2040 (3).

The landscape of breast cancer research is continually evolving, with new insights and innovations emerging rapidly. This series of article collections on the Research Topic “Reviews in Breast Cancer 2023” aimed to showcase cutting-edge research, highlighting recent advances in the field and emphasizing key directions and new possibilities for future investigations.

In this Research Topic, Roheel et al. conducted a systematic review of the global epidemiology of breast cancer, focusing on risk factors. The authors found that lifestyle factors, such as nutrition and exercise, as well as genetic variables, including DNA repair gene polymorphisms and mutations in breast cancer genes (BRCAs), are associated with breast cancer risk. Notably, most of the genetic variability was linked to Asian populations, whereas lifestyle factors were more commonly associated with breast cancer risk in the United States and the United Kingdom. This highlights the differences in demographic, genetic, and lifestyle risk factors across various countries.

These findings are corroborated by Nicolis et al., who emphasized the complex interplay of genetic, lifestyle, and environmental risk factors for breast cancer, noting significant differences between populations. The authors also highlighted the potential of artificial intelligence (AI) to revolutionize personalized breast cancer prevention and detection by tailoring procedures to individual risk factor profiles. Additionally, Chen et al. reported that hepatitis C virus infection is associated with an increased risk of breast cancer.

Abdel-Razeq conducted a systematic review focusing on the oncological safety of less aggressive surgical techniques, including skin-sparing and nipple-sparing mastectomies, for breast cancer patients with mutations in high-penetrance cancer-predisposing genes such as *BRCA1* and *BRCA2*, as well as for unaffected carriers. Additionally, Li et al. provided a systematic review and meta-analysis of axillary treatment in patients with clinically node-negative and sentinel node-positive early breast cancer.

Xu et al. reviewed the role of matrix stiffness in breast cancer progression. Matrix stiffness, which refers to the progressive elastic force exerted by the extracellular matrix on cells, plays a crucial role in regulating various aspects of breast cancer, including tumorigenesis, proliferation, invasion, metastasis, drug resistance, immune evasion, and the growth of breast cancer stem cells. Owing to its significant impact, matrix stiffness has emerged as a potential target for breast cancer treatment. The authors concluded that a deeper understanding of matrix stiffness could pave the way for the development of new therapeutic options for breast cancer.

Several reviews (Ansari et al., Tollens et al., Zhang et al., and Zhang et al.) have concentrated on imaging technologies for breast cancer detection. The authors assessed current modalities, including magnetic resonance imaging (MRI), and examined emerging technologies, such as contrast-enhanced and elastography ultrasound combined with deep learning. Collectively, these reviews highlight the ongoing evolution of imaging technologies and emphasize a clear trend toward integrating traditional imaging techniques with advanced technologies such as contrast enhancement, elastography, and AI-driven analysis. This convergence is expected to enable earlier detection, improve diagnostic accuracy, and ultimately contribute to more favorable patient outcomes.

Beyond imaging, this Research Topic also includes reviews that address other critical aspects of breast cancer, such as novel diagnostic and therapeutic biomarkers (Long et al.), the tumor microenvironment (Akinsipe et al.), surgical interventions (Li et al.), endocrine and immunotherapy (Lan et al., Alaluf et al., and Sharaf et al.), and complementary therapies (Deng et al., and Li et al.). Additionally, Ali-Thompson et al. conducted a bibliometric analysis of HER2-positive breast cancer from 1987 to 2024, offering valuable insights into the research trends and developments in this specific subtype over the past decades.

Early cancer detection is critical for improving overall survival rates, as it enables the initiation of appropriate treatments before metastasis occurs (4). The identification of biomarkers, such as miRNAs, is emerging as a promising strategy for the early diagnosis of breast cancer. Wang et al. conducted a meta-analysis on the association between circulating miR-155 and breast cancer diagnosis and suggested that further large-scale clinical studies on this miRNA are warranted.

The primary cause of death in patients with breast cancer is disease progression due to metastasis and drug resistance. To address this challenge, there is a critical need for reliable molecular biomarkers that can predict disease response. In a meta-analysis and systematic review, Sang et al. found that low absolute lymphocyte counts and elevated neutrophil−lymphocyte ratios were associated with poor outcomes in metastatic breast cancer (mBC) patients. These findings underscore the significant prognostic value of these biomarkers in this patient population.

Trophoblast cell-surface antigen 2 (TROP2) overexpression is associated with aggressive subtypes of breast cancer and drug resistance (5), and its silencing has been shown to reduce tumor growth, underscoring its oncogenic relevance (6). Yao et al. examined the variability of TROP2 expression across different breast cancer subtypes, its correlation with clinicopathological features, and its prognostic and predictive roles. These findings highlight the critical role of TROP2 in tumor dynamics, suggesting that TROP2 represents a compelling therapeutic target.

Wang et al. reviewed the roles and mechanisms of long noncoding RNAs (lncRNAs) in breast cancer progression, metastasis, and drug resistance. They explored lncRNA-based strategies and lncRNA-targeted therapies, emphasizing their potential to enhance the management of breast cancer patients in clinical practice.

In recent decades, significant advancements have been made in the treatment of hormone receptor-positive breast cancer, leading to notable improvements in survival and quality of life. As first-line treatments for hormone receptor-positive mBC patients, cyclin-dependent kinase 4/6 (CDK4/6) inhibitors markedly improve progression-free survival and overall survival. Horani et al. reviewed the literature on the role of CDK4/6 inhibitors in mBC progression. Additionally, Zhang et al. suggested that CDK4/6 inhibitors might also offer therapeutic benefits for HER2-positive breast cancer subtypes, presenting new possibilities for treatment development.

Myeloid differentiation factor 88 (MyD88) overexpression is closely associated with aggressive tumor characteristics, positioning it as a potential prognostic biomarker and therapeutic target. MyD88 plays a crucial role in modulating inflammatory and immune responses, highlighting its impact on the interaction between tumors and the immune system. Zheng et al. analyzed the mechanisms underlying the diverse roles of MyD88 in breast cancer, suggesting that translating these findings into clinical applications holds significant promise for precision medicine approaches, potentially enhancing patient prognosis and therapeutic strategies. Immunotherapy, often utilized in personalized cancer care, strengthens the ability of the immune system to recognize and eliminate cancerous cells.

Alqathama et al. reviewed key immune response-related pathways in breast cancer and discussed how natural compounds can function as immunomodulatory agents that target biomolecular pathways. Some natural compounds have been shown to inhibit immune checkpoints, as well as PD-L1, offering new avenues for therapeutic intervention.

## Conclusions

Overall, the articles compiled in this Research Topic not only consolidate the latest advancements but also provide new insights into breast cancer research. This Research Topic serves as a valuable resource for researchers, clinicians, and policymakers dedicated to enhancing the diagnosis and treatment outcomes for patients with breast cancer.
